# 大力倡导加速康复外科在气管恶性肿瘤介入治疗中的应用

**DOI:** 10.3779/j.issn.1009-3419.2019.01.02

**Published:** 2019-01-20

**Authors:** 洪武 王, 庆好 程, 令煜 孔, 丽 沈

**Affiliations:** 1 100028 北京，煤炭总医院肿瘤内科 Department of Oncology, Beijing 100028, China; 2 100028 北京，煤炭总医院麻醉科 Department of Anesthesiology, Beijing 100028, China; 3 100028 北京，煤炭总医院呼吸内科 Department of Respiratory, Meitan General Hospital, Beijing 100028, China

**Keywords:** 加速康复外科, 支气管镜, 介入治疗, Enhanced recovery after surgery, Bronchoscopy, Interventional therapy

## Abstract

目前加速康复外科（enhanced recovery after surgery, ERAS）已得到全世界越来越多的手术外科及麻醉科的广泛认可，并在结直肠外科、妇科、肝外科、乳腺外科、泌尿外科及脊柱外科等诸多腔镜外科领域得到应用，但在呼吸介入领域应用较少。近年来，越来越多的研究者开始探索ERAS在支气管镜介入治疗中的应用。本文着重从术前准备、麻醉、术中操作、术后观察等多个环节对支气管镜介入治疗的影响方面进行了综述。

## Abstract (en)

Currently, enhanced recovery after surgery (ERAS) has been widely accepted by surgery and anesthesiology all over the world, and applied in colorectal surgery, gynecology, liver surgery, breast surgery, urology and spinal surgery. But ERAS are rarely used in the field of interventional bronchoscopy. In recent years, more and more researchers have begun to explore the application of ERAS in bronchoscopic interventional therapy. This article discussed that preoperative preparation, anesthesia, intraoperative operation, postoperative observation and other aspects can influence interventional bronchoscopy.

恶性气管肿瘤可分为原发性和转移性两种。原发性气管肿瘤常见的有鳞癌、腺样囊性癌、类癌、及腺癌等。转移性肿瘤可来自全身各处，直接侵犯气管的可来自食管、纵隔、甲状腺、胸腺等肿瘤。远处转移的肿瘤包括上呼吸道肿瘤、消化道肿瘤、乳腺癌、肾细胞癌、转移性黑色素瘤以及淋巴瘤等^[1, 2]^。由于肿瘤所处的部位比较特殊，如不及时处理，有可能引起窒息等严重并发症。因此，对肿瘤堵塞比较严重的病变，需按急症处理，传统治疗方法如手术、放疗、化疗等，可能力不从心。而介入治疗可尽快打通气道，消除气道梗阻，对改善生存质量、延长生命有重要意义。由于病变部位和性质不同，介入治疗方法各异，疗效也大不相同。近年来，作者已牵头出台了多个专家共识^[3-5]^，希望对恶性肿瘤的治疗起到规范化作用。

加速康复外科（enhanced recovery after surgery, ERAS）于1997年由丹麦Kehlet教授首次提出并应用于临床，其核心是以循证医学证据为依据，多学科合作，优化围手术期处理措施，改善患者预后，缩短围术期住院时间，降低医疗费用，减少并发症^[6, 7]^。21年来，这种多学科、多模式围术期康复干预的理念已得到全世界越来越多的手术外科及麻醉科的广泛认可，并在结直肠外科、妇科、肝外科、乳腺外科、泌尿外科及脊柱外科等诸多腔镜外科领域得到应用^[8-10]^。近年来，中国医师协会麻醉学医师分会、中华医学会骨科学分会关节外科学组、普通外科、麻醉科、胸心外科和神经外科等陆续发表了ERAS中国专家共识。

近年来，作者也试图将ERAS理念引入到呼吸介入治疗中，无奈缺少理论依据，不能在呼吸介入治疗中推行。作者根据自己多年的临床经验，参照相关知识，希望能抛砖引玉，尽快在该领域建立自己的体系，是否需要创立一个新的名词——加速康复支气管镜（enhanced recovery after bronchoscopy, ERAB）以区别于ERAS，有待商榷。

针对恶性气管肿瘤的各个环节，作者认为有必要从术前准备、麻醉、术中操作、术后观察等多个环节做好细致的ERAB，这些工作需要通过多学科医护共同完成，才能达到预期目的。呼吸内镜医师是实施ERAB的关键，麻醉科医师和护士应积极参与内镜术前评估和术前准备。病房护士对围手术期评估和康复也有重要作用。

ERAB的实施：实施ERAB的措施，包括术前的宣教、饮食准备，术中麻醉方式和药物的选择、影像准备、凝血功能检测、血象及血型鉴定、病毒性感染指标测定等。

## 1 术前评估及宣教

术前评估患者呼吸介入治疗风险，特别要认真评估气道阻塞的程度和部位，以及术中可能采用的麻醉方法和治疗方法。

根据中央型气道八分区方法^[11]^，气管肿瘤主要位于Ⅰ区、Ⅱ区、Ⅲ区和Ⅳ区。经过大数据分析，各区均以鳞癌最常见，Ⅰ区还易受甲状腺癌侵犯，Ⅱ区、Ⅲ区其他常见的肿瘤还有腺样囊性癌和食管癌。Ⅳ区第二位的肿瘤是腺样囊性癌。

根据肿瘤与管壁的关系，可分为四种类型^[2]^：①管内型，肿瘤局限在管腔内，则称为内生型即腔内型；②管外型，单纯因气管外肿瘤生长压迫致气管狭窄，且无腔内组织和管壁受损，则称为外压型即腔外型；③管壁型，沿管壁匍匐性生长，基底较宽，管壁较厚，可致管腔狭窄；④混合型，肿瘤有腔内生长，也有管壁、管外累及即称为混合型。混合型肿瘤常源于毗邻气道的组织、侵犯气道壁并进而侵入管腔。对有明确肿块的气管癌，术前还应行增强计算机断层扫描（computed tomography, CT）扫描，以确定肿瘤是富血管还是乏血管。对富血管的肿瘤术前应行靶血管栓塞，以减少术中出血的风险^[12]^。

恶性气道狭窄严重程度的分级通常依据管径的狭窄程度（%）进行分级，管径的狭窄程度=狭窄管径/正常管径×100%。狭窄的严重程度分为5级。1级为狭窄程度≤25%，2级为26%-50%，3级为51%-75%，4级为76%-90%，5级为91%-100%^[2]^。

一般认为1级为轻度狭窄，可有轻度咳嗽等症状；2级、3级为中度狭窄，可有咳嗽、气短等症状；4级、5级为重度狭窄，则有严重呼吸困难，可出现三凹征、发绀，甚至窒息死亡。呼吸困难的程度主要取决于狭窄气道的直径大小。一般情况下，当肿瘤堵塞或压迫引起气管狭窄程度 > 50%时，患者会出现明显的呼吸困难。

术前宣教被认为是围术期不可或缺的一部分。内镜和麻醉医生不仅要通过合适的沟通方式缓解患者的焦虑情绪，还要为患者制定术前镇静镇痛药物运行和饮食方案。根据ERAS的要求，禁食固体食物和禁饮时间分别缩短为6 h和2 h，并且在术前2 h口服400 mL碳水化合物，有助于减轻患者术前饥饿感，降低术中胰岛素抵抗，促进术后快速康复^[10]^。这比传统的术前禁食12 h至少缩短了6 h的时间，也就是至少减轻了患者6 h的饥饿感。

术前应提前建立输液通道，应用多功能心电血压监护仪进行无创血压、心电、呼吸、血氧饱和度监测。应用BISS监测脑电活动，便于术中准确用药。

## 2 麻醉

呼吸介入的麻醉至关重要。如气管堵塞较轻（气管狭窄≤50%）、病情较轻，可耐受气管镜诊治，可选用局麻或监控静脉麻醉下操作。但如果气管堵塞较重（气管狭窄 > 70%）、病情也较重，则需在监控静脉麻醉或全凭静脉麻醉下进行。

局部麻醉：局部麻醉是应用局部麻醉药暂时阻断身体某一区域的神经传导而产生麻醉作用，简称局麻。局麻简便易行，安全性大，能保持患者清醒，对生理功能干扰小，并发症少，适用于较表浅局限的中小型手术。临床上支气管镜检查和治疗应用的局部麻醉方法有喉头喷雾法、超声雾化法、压缩泵雾化法、气管内滴药法、含漱法、环甲膜穿刺注药法。常用局麻药物为2%利多卡因。利多卡因气雾剂：作为一种新型麻醉剂，在气管镜检查中经鼻吸入操作简单、使用方便、起效快、麻醉效果好、安全性高，是气管镜检查中值得推广的新麻醉方法。

局部麻醉+监控麻醉：MAC技术即监控麻醉（monitored anesthesia care, MAC），指麻醉医师参与局麻患者的监测和/或对接受诊断性或治疗性操作的患者使用镇静-镇痛药物，以解除患者焦虑及恐惧情绪，减轻疼痛和其他伤害性刺激，提高围术期的安全性和舒适性。

在支气管镜介入治疗的检查过程中应用一定量的静脉镇静镇痛药使患者有一短暂睡眠过程，检查治疗完毕后，患者能迅速清醒，对整个检查过程无记忆、无痛苦感觉，避免了常规支气管镜检查时患者应激反应造成的生理、心理上的不适，使检查得以顺利完成，有利于提高检查效果及复查率。

MAC期间麻醉医师应使用镇静、镇痛、麻醉和心血管药物为患者在舒适和安全之间提供一个最佳的平衡。术中应常规作血氧饱和度和无创血压的监测防止低氧血症和低血压的发生。对老年患者或已有心肺功能减退的患者应作心电图监测，同时要不断对镇静状态进行评分，避免镇静、麻醉过深。

MAC常用药物有阿片类止痛药芬太尼及其家族，神经安定镇静药咪达唑仑、静脉麻醉药异丙酚和依托咪酯等。

全凭静脉麻醉：全凭静脉麻醉即在静脉麻醉诱导后，采用多种短效静脉麻醉药复合应用，间断或连续静脉注射维持麻醉的方法。全凭静脉麻醉是目前无痛支气管镜介入治疗检查的主要麻醉方法。由于全麻会产生心血管系统的抑制、对呼吸的影响、肌松效应等，所以需要专业麻醉医师来实施^[13]^。

静脉给予东莨菪碱0.3 mg或阿托品0.5 mg。诱导前吸入纯氧5 min。根据患者心肺功能评估情况给予静脉诱导。诱导药物依次为：咪达唑仑2 mg-3 mg，舒芬太尼5 µg-10 µg，异丙酚1 mg/kg-2 mg/kg或者依托咪酯0.1 mg/kg-0.2 mg/kg，罗库溴铵0.6 mg/kg-0.9 mg/kg。诱导后，置入喉罩或气管导管，或在电视显示器引导下置入硬质气管镜。术中维持用药丙泊酚4 mg/（kg∙h）-6 mg/（kg∙h），瑞芬太尼0.1 µg/（kg∙min）-0.2 µg/（kg∙min），术中间断追加舒芬太尼。治疗结束前30 min，静脉给予地塞米松10 mg或甲泼尼龙80 mg。

全麻过程中的通气管理：呼吸支持常规采用高频喷射呼吸机，将喷射管连接硬质气管镜侧孔或通过L形接头连接喉罩或气管插管进行高频喷射通气。高频喷射呼吸机可产生高频率、低潮气量、低气道压。高频通气循环干扰少，不影响自主呼吸。经过大量的临床实践，目前用常频：频率20次/min-40次/min，驱动压为0.25 Mpa-0.35 Mpa，吸呼比1:1。这样可使每次喷射的时间较长，潮气量较大，气流经过的距离更远而有利于肺泡内气体交换，减少肺内功能性分流，纯氧通气可使吸入氧浓度 > 60%。术中患者SpO_2_ < 90%则撤掉高频喷射呼吸机换接麻醉机，通过麻醉机快速充氧手法控制呼吸。治疗结束退出硬质气管镜或气管插管，根据患者恢复情况置入喉罩或者气管内导管，连接麻醉机行机械通气排出体内CO_2_，直至患者苏醒。

高频通气时由于支气管镜的操作、高频喷射的气流方向改变、氩等离子治疗时消耗氧气等原因限制气体的进出，使吸入氧气的浓度随着输送逐渐递减，所以肺泡内的实际FiO_2_低于吸入的100%氧浓度，因此应给予患者吸入100%纯氧，复合自主呼吸以减少低氧血症的发生。很多患者肺癌晚期合并肺炎、肺纤维化等疾病，耐受性差，治疗时易出现低氧血症且SpO_2_难以升高，可通过持续应用麻醉机快速直接充氧手法控制呼吸，必要时暂停治疗。对于SpO_2_持续低下的患者，术者应对气道内进行有效的负压吸引，减少烟雾、血液、血凝块和碎屑对肺通气和弥散的影响。吸引后要充分膨肺，减少肺泡无效腔量。

部分患者在高频喷射通气时存在CO_2_潴留，主要是由于患者的肺储备功能降低、气管阻塞严重、手术时间、高频通气的次数和气体交换时间等引起。应通过血气分析结果来调整呼吸次数和驱动压。

## 3 术中操作

常用的进镜方法有经鼻、经口、经气管插管、经气管切开造口、经喉罩、经硬质镜（rigid bronchoscopy, RB）等途径进入。

喉罩：操作简单，置入容易，呼吸道损伤小，对循环系统无明显影响，适用于高血压，合并心血管疾病的患者，应用于支气管镜检查有良好的效果。操作中支气管镜可以从三通连接管进入气道进行检查，通过三通连接管侧管接呼吸机进行控制呼吸，由于喉罩将会厌挑起固定，加上肌松药的使用使呼吸得到控制，声门开放良好，使插镜顺利，而且患者意识消失，无呛咳及体动反应，镜检医师可以从容进行检查和治疗，既不影响操作又能安全有效的控制呼吸，操作时间也不再受限，充分显示了其在支气管镜麻醉中的优越性。对于声门下7 cm以内的病变，包括气管中、上段及声门下病变，可选择喉罩。

气管插管：对气管堵塞较重或全身情况较差、不能耐受局麻的患者，可行气管插管。一般选择直径7.5 mm-9 mm的气管插管，在应用软性支气管镜进行介入治疗时一般不会遇到因气道压力增高导致手术中断的情况。因此对于声门下7 cm以下的病变，包括气管下段、隆突及左右主支气管的病变一般可采用气管插管的方法。

硬质气管镜^[14]^：对气管任何部位堵塞较重或全身情况较差、不能耐受局麻的患者，均可选择RB。由于其外径小于气管内径，治疗时气道是开放的，因此选择静脉全麻。术中通过丙泊酚和瑞芬太尼维持，间断追加舒芬太尼，使患者在治疗过程中没有明显的体动反应，避免呛咳影响治疗。麻醉诱导后可以不再追加肌松药，术中保留微弱的自主呼吸，这样利于CO_2_的排出。

传统RB插入法需5 min-10 min，而作者将插入法改良后，5 s-10 s内可快速插入，大大缩短了时间。在软质气管镜引导下插入RB^[1]^：将软镜插到硬质镜镜鞘内，距鞘尖端部5 mm-10 mm，切勿将软镜伸出镜鞘。经口插入镜鞘，沿舌背前行，看到会厌后将会厌挑起，将镜鞘侧转90度，进入声门，然后再将镜身转正。随即镜鞘末端连接三通管，对接高频喷射呼吸机，调至呼吸频率20次/min-40次/min（低频），维持患者血氧饱和度在100%。如为声门部或声门下肿瘤，硬质镜前端斜面跨过声门即可，由助手固定硬质镜进行操作。在不停呼吸机的情况下经过镜鞘后孔进行各种操作。若操作一段时间后，高频喷射通气不能维持足够的氧饱和度，可改用麻醉机，必要时用手动式球囊按压，将血氧饱和度维持在100%以上时，再继续进行操作。

大多数硬质镜需与电子支气管镜相结合进行介入治疗^[15]^。两种镜种结合应用，可缩短每次操作时间，减少检查次数，疗效更好。

如果因各种原因RB无法插入，亦可换用气管插管或喉罩，再结合电子支气管镜进行介入治疗。

呼吸内镜医生要熟练应用各种仪器设备。呼吸内镜下有许多技术和设备，目前常用的有热消融（激光、电刀、氩气体凝固术）、冷消融（冻融或冻切）、机械性切除（硬质镜铲除术和钳取术）和气道扩张（支架置入或硬质镜扩张）技术，这些技术的目的是快速达到通畅气道、改善通气和防止窒息的作用，这与加速康复外科理念一致^[16-18]^。每一种技术和设备都有特殊的要求，需熟练掌握。如热消融热备，术前需连好电极、调整好能量功率，激光需准备好光导纤维和调整好激光能量，支架需选好型号等。

术中还需根据不同的病变采取不同的技术^[1, 14]^。如管内病变，可采用电圈套器、APC、冻取等，对管壁病变可采取铲切、APC、激光等，对管外压迫病变，可采用内支架置入等技术。如何序贯或同步采用这些技术，就是一种艺术问题，即整合治疗。

支架置入术可起到持续扩张气道的作用，亦可应用堵瘘^[5]^。如何选择最合适的支架需考虑术者的经验、所需费用、病灶的结构以及患者病情。中央型气道的八分区方法对选择气道支架的形状有重要指导意义。对Ⅰ区、Ⅵ区、Ⅷ区病变适合放置直筒型支架，而对隆突附近的Ⅱ区、Ⅲ区、Ⅳ区、Ⅴ区、Ⅶ区则适合放置分叉支架（L或Y形）。硅酮支架需要全麻并在硬质镜下放置。

术中内镜医生和助手间的配合默契程度，对手术进程也有重要作用。

麻醉师还要根据患者的情况不断调整用药，维持患者持续麻醉状态和稳定的生命体征。在临近手术结束时，要及时减药，必要时应用拮抗药，迅速让患者苏醒，恢复自主呼吸状态，并在5 min左右拔出硬质镜，洗净口腔内的分泌物，待患者稳定后送回普通病房，并与病房护士交接清楚，说明术中情况和术后需注意事项。

若患者麻醉后不能很快清醒，或仍有严重气道阻塞或呼吸衰竭，则需行气管插管或喉罩，转至重症监护室（intensive care unit, ICU），继续观察处理。直至病情稳定后方能拔出气管插管，转出ICU。

## 4 术后康复

ERAB术后处理强调“早发现、早处理”并发症。术后需密切观测生命体征的变化和每一种治疗的并发症。术后宜采取半卧位，不要去枕平卧位。术后2 h-3 h可下床活动和经口进食。

ERAS在腔镜领域已取得丰富经验，呼吸内镜医师应尽快迎头赶上，尽快普及这方面的知识。未来ERAB需要我们内镜大夫、麻醉大夫、手术室护士和病房护士、患者及其家属共同努力，以提高患者生存质量、改善患者预后，减少并发症，缩短住院时间和降低住院费用。通过多学科资源整合，促进ERAB在呼吸内镜领域形成和发展。目前倡导多学科协同诊疗模式，以患者为中心，依托多学科团队，制定规范化、个体化、连续性的综合诊疗方案，麻醉科医生积极主动参与，对现代医学ERAB的实施有着重要的意义。
